# *In Vitro* Cytotoxicity Evaluation of Plastoquinone Analogues against Colorectal and Breast Cancers along with *In Silico* Insights

**DOI:** 10.3390/ph15101266

**Published:** 2022-10-14

**Authors:** Halilibrahim Ciftci, Belgin Sever, Nilüfer Bayrak, Mahmut Yıldız, Hatice Yıldırım, Hiroshi Tateishi, Masami Otsuka, Mikako Fujita, Amaç Fatih TuYuN

**Affiliations:** 1 Department of Drug Discovery, Science Farm Ltd., Kumamoto 862-0976, Japan; 2 Medicinal and Biological Chemistry Science Farm Joint Research Laboratory, Faculty of Life Sciences, Kumamoto University, Kumamoto 862-0973, Japan; 3 Department of Molecular Biology and Genetics, Koc University, 34450 Istanbul, Turkey; 4 Department of Pharmaceutical Chemistry, Faculty of Pharmacy, Anadolu University, 26470 Eskisehir, Turkey; 5 Department of Chemistry, Faculty of Science, Istanbul University, Fatih, 34126 Istanbul, Turkey; 6 Chemistry Department, Gebze Technical University, Gebze, 41400 Kocaeli, Turkey; 7 Department of Chemistry, Faculty of Engineering, Istanbul University-Cerrahpasa, Avcılar, 34320 Istanbul, Turkey

**Keywords:** colorectal cancer, breast cancer, plastoquinone, NCI-60, growth inhibition, cytotoxicity, apoptosis, DNA binding, pharmacokinetic determinants

## Abstract

Colorectal cancer (CRC) and breast cancer are leading causes of death globally, due to significant challenges in detection and management. The late-stage diagnosis and treatment failures require the discovery of potential anticancer agents to achieve a satisfactory therapeutic effect. We have previously reported a series of plastoquinone analogues to understand their cytotoxic profile. Among these derivatives, three of them (**AQ-11**, **AQ-12**, and **AQ-15**) were selected by the National Cancer Institute (NCI) to evaluate their *in vitro* antiproliferative activity against a panel of 60 human tumor cell lines. **AQ-12** exhibited significant antiproliferative activity against HCT-116 CRC and MCF-7 breast cancer cells at a single dose and further five doses. MTT assay was also performed for **AQ-12** at different concentrations against these two cells, implying that **AQ-12** exerted notable cytotoxicity toward HCT-116 (IC_50_ = 5.11 ± 2.14 μM) and MCF-7 (IC_50_ = 6.06 ± 3.09 μM) cells in comparison with cisplatin (IC_50_ = 23.68 ± 6.81 μM and 19.67 ± 5.94 μM, respectively). This compound also augmented apoptosis in HCT-116 (62.30%) and MCF-7 (64.60%) cells comparable to cisplatin (67.30% and 78.80%, respectively). Molecular docking studies showed that **AQ-12** bound to DNA, forming hydrogen bonding through the quinone scaffold. *In silico* pharmacokinetic determinants indicated that **AQ-12** demonstrated drug-likeness with a remarkable pharmacokinetic profile for future mechanistic anti-CRC and anti-breast cancer activity studies.

## 1. Introduction

Colorectal cancer (CRC), the third most common cancer type and the fourth leading cause of cancer-related death, comprises nearly 10% of all annually diagnosed tumors across the world [1,2,3,4]. Male gender, old age, dietary habits, and environmental factors affect the pathogenesis of CRC as well as the genetic background [5]. In spite of advancements in CRC screening and treatment options such as surgery, radiotherapy, local ablative therapy, chemotherapy, targeted therapy, and immunotherapy, most of cases in particular diagnosed at an advanced stage with metastases, result in subsequent cancer-related deaths. The size, stage, and metastasis of tumor whether the therapy will be curative or palliative. Among CRC treatment options, chemotherapy incorporates single-agent therapy (primarily fluoropyrimidine, oxaliplatin, irinotecan, and capecitabine) or combined regimens of these agents. Targeted therapy is another approach, which has been reported to extend survival of patients with CRC. However, the resistance and toxicity problems restrict the success of the therapy. Therefore, there is an urgency to find more effective and safer drugs [2,6].

The history of breast cancer was shown to be a risk factor for CRC in several studies, implying low or high relative risks. These risks were considered due to the common etiologic factors associated with the development of both cancers and administration of antihormone drugs in breast cancer treatment, which alter sex hormone levels and contribute to the development of CRC [7,8,9,10,11]. New efficacious therapeutic options have been also developed for the battle with breast cancer related to its clinical stage, histopathologic properties, and biomarker profiling. These options include traditional, personalized, neoadjuvant, and targeted therapies. The treatment still remains limited mainly in the breast cancer metastasis owing to heterogeneity of the disease, acquired and primary resistance, and toxicity problems during the treatment. New agents should be also developed to overcome or prevent these problems in breast cancer treatment [12,13,14].

Much effort to design and discover efficient and safe drug candidates led to identifying several hit compounds and analogues of natural products. *In silico* analyses were exploited to improve molecules with greater potential efficacy to cope with the adverse toxicological outcomes by emphasizing physicochemical parameters [15]. In addition, the study of the structure–activity relationship (SAR) has provided valuable information on the design of safe drug candidates with continuity about how structural changes can improve potency and bioavailability [16].

**Figure 1:**
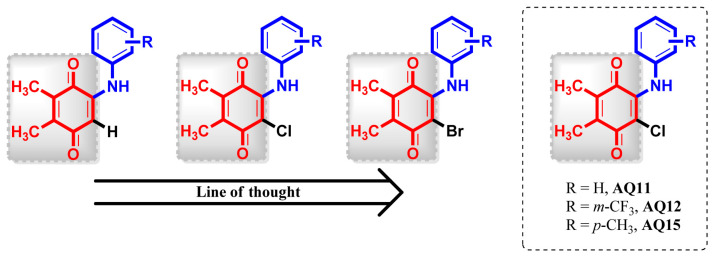
Line of thought. Structures of target molecules (**AQ-11**, **AQ-12,** and **AQ-15**).

1,4-Quinones have been explored as attractive anticancer hit molecules to their multitargeted mode of actions [17,18,19,20,21,22]. In this field, a small library of amino-quinones based on bioactive natural products (fifty Plastoquinone (PQ) [23,24,25] and thirty-four LY83583 analogues [26,27]) that specifically target leukemia cancer cell lines, in the period 2017–2021, was generated for the purpose of discovering the SAR of various substituents in aminoquinones for their further mechanistic anticancer potential. Knowing that the PQ analogues are active toward some cancer cell lines, and considering our previous findings that demonstrated greater activity by the introduction of a chlorine atom in the quinone moiety, we also designed and evaluated the effect on inhibitory activity against some cancer cell lines caused by replacing the chlorine with a bromine atom in PQ analogues [28]. Regarding all PQ analogues, including halogenated (brominated and chlorinated) and non-halogenated analogues, a major breakthrough was the discovery of **AQ-11, AQ-12**, and **AQ-15** [25], as illustrated in Figure 1. These analogues showed consistent growth-inhibitory activities with low IC_50_ value against K562 human chronic myelogenous leukemia (CML) cell line and low toxicity toward human peripheral blood mononuclear cells (PBMCs) (healthy) (Table 1) [25].

Considering the encouraging results of the target PQ analogues, further studies were assessed aiming at the identification of new analogues for their antiproliferative activity against HCT-116 human CRC and MCF-7 human breast cancer cell lines. In addition, apoptosis-inducing activity in both cell lines, DNA binding characteristics, and a number of pharmacokinetic descriptors of the most effective anticancer analogue were examined.

## 2. Results

### 2.1. Anticancer Activity Assessment

#### 2.1.1. *In Vitro* Screening of Tumor Cell Growth Inhibition at One Dose

Continuing our efforts on anticancer drug discovery, most effective PQ analogues from our previous study [25] were submitted to the National Cancer Institute (NCI) of Bethesda within the Developmental Therapeutics Program (DTP) for their *in vitro* anticancer activity with the protocol of the Drug Evaluation Branch, NCI. A single dose (10 µM) of all tested PQ analogues was used in the panel of 60 human cancer cell lines, including nine tumor subpanels, namely: leukemia, lung, CRC, central nervous system (CNS), melanoma, ovarian, renal, prostate, and breast cancer cell lines [29,30,31]. The *in vitro* growth inhibition and lethality were ascertained as percentages as follows: growth inhibition (G%) (values between 0 and 100) and lethality (values less than 0). Herein, three PQ analogues (**AQ-11**, NCI: D-827199/1; **AQ-12**, NCI: D-827200/1; and **AQ-15**, NCI: D-827201/1) were selected by the NCI for *in vitro* disease-oriented human-cell-screening panel assay.

The results of each tested PQ analogue were reported in terms of percent growth inhibition (GI% = 100 − G%) and lethality [32] (Table 2) and were also depicted as bars in the single-dose mean graphs (Supplementary Materials, Figures S1–S3). Overall, consistent with the previous data, PQ analogues showed the most notable anticancer activity against leukemia cancer cell lines. **AQ-11** and **AQ-15** were found ineffective against the other cancer cell lines except for significant anticancer effects of **AQ-11** on MDA-MB-231 breast cancer cell line with 84.65% inhibition percent. On the other hand, NCI-60 data suggested that **AQ-12** revealed prominent anticancer activity toward the subpanel cell line of CRC (HCT-116 cells, 66.14% inhibition; SW-620 cells, 82.93% inhibition) and breast cancer (MCF-7 cells, 64.64% inhibition; MDA-MB-231 cells, 81.04% inhibition). Additionally, this analogue also showed promising anticancer effects against NCI-H522 lung cancer cells.

#### 2.1.2. *In Vitro* Full-Panel Five-Dose 60-Cell Lines Assay

From the single-dose assay data from the NCI screen, **AQ-12** was selected as a lead PQ analogue because of its pronounced anticancer selectivity compared with other PQ analogues. **AQ-12** exhibited the threshold inhibition criterion in the single-dose screening and was qualified for the evaluation in the full-panel five-dose *in vitro* anticancer screening at 10-fold dilutions in the range 0.01–100 µM. Three response parameters (50% cell growth inhibition (GI_50_) (growth inhibitory activity), total cell growth inhibition (TGI) (cytostatic activity), and 50% cell death (LC_50_) (cytotoxic activity)) [33] were used to establish biological potential of the tested **AQ-12**. The GI_50_ is an indicative concentration at 50% growth inhibitory activity, whereas TGI reflects total growth inhibition, and LC_50_ is an indicative concentration at which 50% of cancer cells are killed. In this assay, three parameters were calculated for each cell line from log concentration versus percent growth inhibition curves on nine panels of human cancer cell lines to generate dose response curves [31,34]. GI_50_ is the concentration of the test drug where 100 × (T − T0)/(C − T0) = 50. Herein, T explains the optical density of the test well after a 48 h period of treatment with the test drug; T0 explains the optical density at time zero; ultimately, C is the control (nondrug) optical density. The “50” is called the GI50PRCNT, a T/C-like parameter that can have values from +100 to −100. The TGI is the concentration of test drug where 100 × (T − T0)/(C − T0) = 0. LC_50_ is the concentration of the drug where 100 × (T − T0)/T0 = −50 [31].

**Figure 2:**
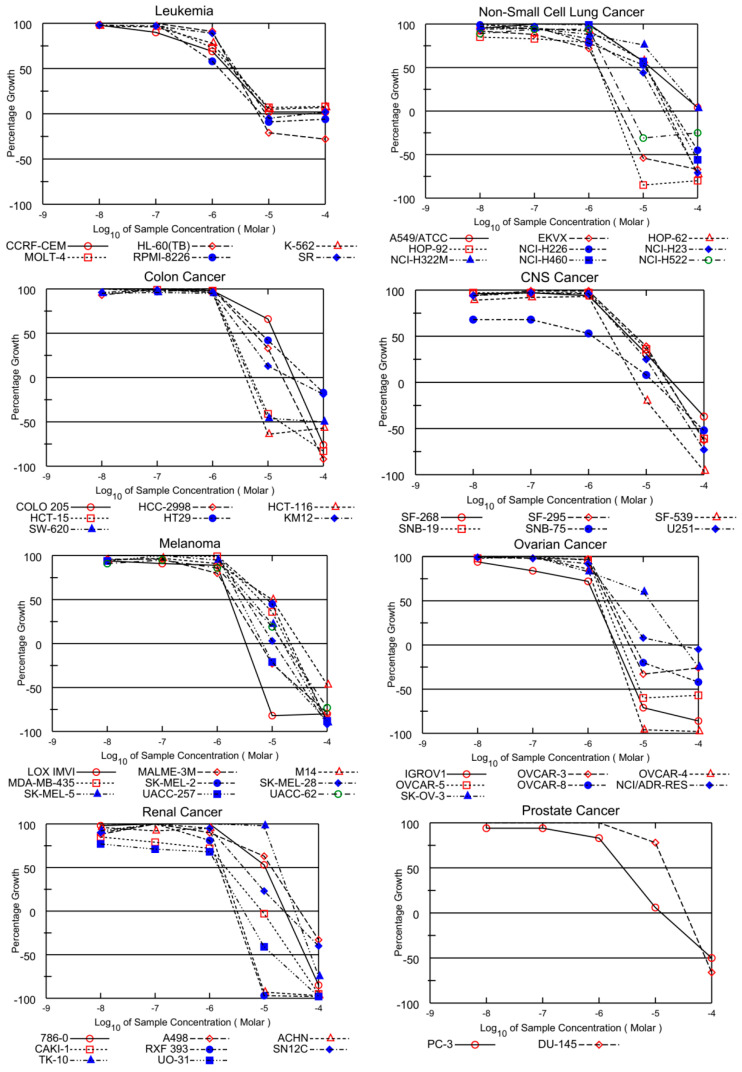

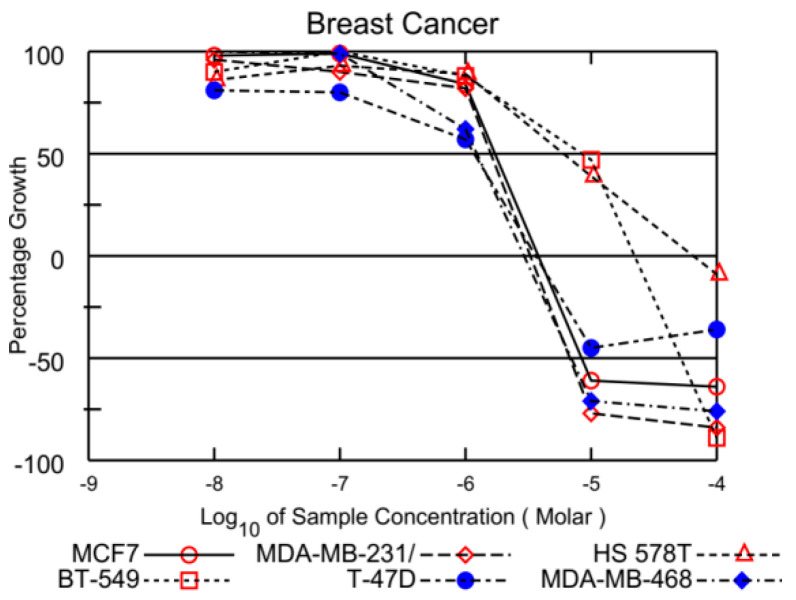
Graphical presentation of growth inhibition of the PQ analogue **AQ-12** at five dose concentrations (0.01, 0.1, 1, 10, and 100 µM) after 48 h based on SRB assay at NCI.

The GI_50_, TGI, and LC_50_ (in µM) values against subpanel cell lines are illustrated in Table 3, indicating that **AQ-12** displays high anticancer activity against all leukemia cell lines with GI_50_ values ranging from 1.32 to 2.59 µM. This compound also demonstrated superior cytotoxic activity against HL-60(TB) and RPMI-8226 cell lines with TGI values 6.54 and 7.32 µM, respectively. On the other hand, LC_50_ values were more than 100 µM against the entire panel of cancer cell lines. Moderate cytotoxicity was recorded against non-small cell lung cancer cells, except for EKVX cell line with a GI_50_ value of 1.49 µM, HOP-92 cell line with a GI_50_ value of 1.51 µM, and NCI-H522 cell line with a GI_50_ value of 2.24 µM. Moreover, good TGI values ranging from 3.04 to 32.20 µM were obtained against lung cancer cell lines. Additionally, **AQ-12** showed high activity with GI_50_ values ranging from 1.93 to 2.20 µM against some CRC cell lines (HCT-116 cells GI_50_ = 1.93 µM; HCT-15 cells GI_50_ = 2.20 µM; SW-620 cells GI_50_ = 2.09 µM). It also possessed notable TGI values against these three CRC cell lines in the range of 3.99–5.05 µM. Moderate cytotoxicity was recorded against CNS cancer with GI_50_ values ranging from 4.45 µM to 6.55 µM against most of the tested cancer cell lines. Moreover, important TGI values ranging from 13.60 to 28.60 µM were detected against CNS cell lines. **AQ-12** demonstrated pronounced cytotoxicity with GI_50_ values ranging from 3.36 to 9.96 µM, except for three cell lines (LOX IMVI, MALME-3M, and UACC-257 cells). Concerning the TGI values of **AQ-12**, the most prominent TGI value was observed with the LOX IMVI melanoma cell line (TGI = 3.32 µM). Furthermore, this analogue demonstrated notable anticancer activity against the entire panel of ovarian cancer cell lines with GI_50_ values ranging from 1.30 to 3.16 µM, as shown in (Table 3). Its TGI values ranged from 2.97 to 50.70 µM against all tested cancer cell lines. Finally, its LC_50_ values were found as 7.15, 5.58, and 8.61 µM against IGROV1, OVCAR-4, and OVCAR-5 cell lines; whereas, with regard to the lethality (LC_50_ values), **AQ-12** showed values exceeding 100 µM toward the other panel cancer cell lines. This analogue exhibited remarkable anticancer activity against the renal, prostate, and breast cancer cell lines with GI_50_ values ranging from 1.17 to 2.67 µM. Accepted pronounced TGI values were recorded against ACHN renal (TGI = 3.19 µM), RXF 393 renal (TGI = 2.85 µM), UO-31 renal (TGI = 4.18 µM), MCF-7 breast (TGI = 3.79 µM), MDA-MB-231 breast (TGI = 3.28 µM), T-47D breast (TGI = 3.60 µM), and MDA-MB-468 breast (TGI = 2.94 µM) cancer cell lines, together with PC-3 prostate (TGI = 12.80 µM) cancer cell line. All the five-dose response curves of **AQ-12** against the full panel of 60 human cancer cell lines are presented in Figure 2 and Table 3, which include nine tumor subpanels, namely: leukemia, melanoma, CRC, lung, CNS, ovarian, renal, prostate, and breast cancer cell lines.

#### 2.1.3. Cell Viability Assay on CRC and Breast Cancer Cells

**AQ-12** was found to possess a superior sensitivity profile toward HCT-116 CRC and MCF-7 breast cancer cell lines with a higher growth inhibitory percent compared to **AQ-11** and **AQ-15**. **AQ-12** also displayed sensitivity toward MDA-MB-468 breast and NCI-H522 lung cancer cell lines. However, **AQ-11** also presented significant growth inhibitory effects on MDA-MB-468 breast cancer cells, restricting the selectivity of **AQ-12** against this cell line, and GI_50_, TGI, and LC_50_ parameters were found very high against NCI-H522 lung cancer cells. Satisfactory results obtained from both single and five doses of NCI-60 screening toward HCT-116 CRC and MCF-7 breast cancer cells encouraged us to further investigate the anticancer effects of **AQ-12** against these two cell lines by MTT (3-(4,5-dimethyl-2-thiazolyl)-2,5-diphenyltetrazolium bromide) assay at five dose concentrations (1, 3, 10, 30, and 100 μM) in comparison with cisplatin, the reference agent. 

Cisplatin, a metallic coordination compound leading to DNA damage and subsequently apoptosis induction in cancer cells, is one of the most important chemotherapeutic agents, which has been approved for the treatment of different fatal cancer types, including CRC and breast cancer [35,36,37,38,39,40,41].

**Figure 3:**
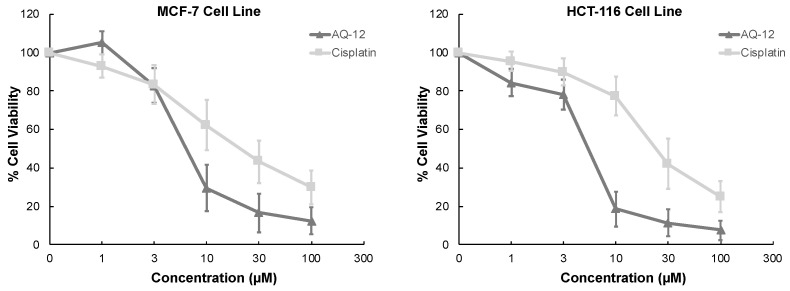
The cytotoxic effects of **AQ-12** and cisplatin at varying concentrations (1, 3, 10, 30, and 100 μM) on MCF-7 and HCT-116 cells based on MTT assay. All descriptive data are expressed as the mean ± standard deviation (SD). All experiments were repeated three times.

Results indicated that **AQ-12** showed cytotoxic effects on HCT-116 and MCF-7 cells with IC_50_ values of 5.11 ± 2.14 μM and 6.06 ± 3.09 μM when compared with cisplatin (IC_50_ = 23.68 ± 6.81 μM for HCT-116 cells and 19.67 ± 5.94 μM for MCF-7 cells). The definite decline in percentage of viable cells was detected between 3 and 10 μM after **AQ-12** exposure while a similar decline was observed between 10 and 30 μM after cisplatin implementation (Figure 3, Table 4).

#### 2.1.4. Cell Death Investigation

**Figure 4:**
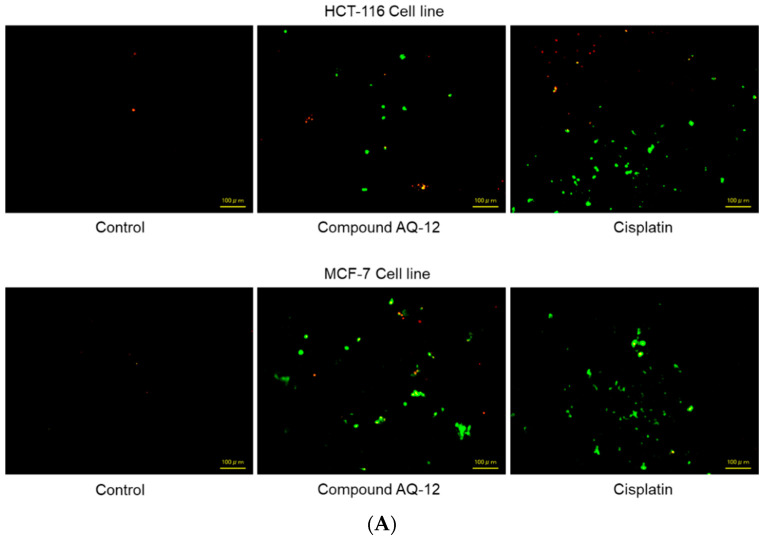

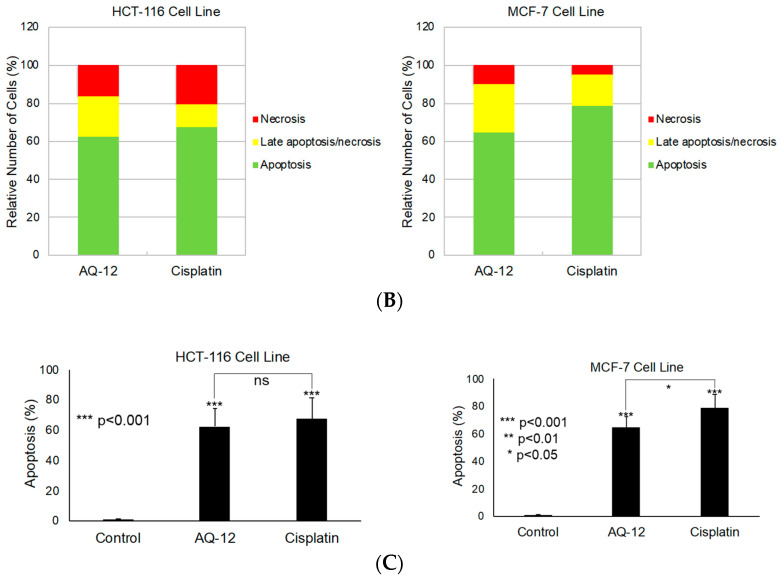
(**A**) Alteration of HCT-116 and MCF-7 cells after treatment with IC_50_ concentration of the control (DMSO), **AQ-12,** and cisplatin (**B**) for 12 h. The percentage of apoptosis, late apoptosis/necrosis, and necrosis (green, yellow, and red, respectively) cells (**C**) was determined by analyzing 100 randomly selected stained cells in each experiment (ns: not statistically significant). Data from three independent experiments were expressed as means ± standard deviation and *p* values were determined using Student’s test.

Based on significant anticancer activity results of **AQ-12** on HCT-116 and MCF-7 cells, we also further investigated potential effects of this compound on apoptosis in both cell lines using the annexin V/ethidium homodimer III staining procedure, which was observed by fluorescence microscopy, indicating apoptosis, necrosis or late apoptosis, and necrosis with green, yellow, and red staining, respectively (Figure 4A). **AQ-12** induced apoptotic behavior of HCT-116 cells (62.30%) in a similar manner with cisplatin (67.30%). This compound exhibited 21.30% late apoptotic/necrotic and 16.40% necrotic effects in HCT-116 cells compared to cisplatin (12.30% and 20.40%, respectively) (Figure 4B). The difference of apoptosis induction between **AQ-12** and cisplatin treatment in HCT-116 cells was found not significant, contrary to that of MCF-7 cells, which was found significant (Figure 4C). **AQ-12** enhanced apoptosis in MCF-7 cells significantly with 64.60% when compared with cisplatin (78.80%). **AQ-12**-treated MCF-7 cells underwent late apoptosis/necrosis and necrosis with 25.30% and 10.10%, respectively when compared with cisplatin (16.40% and 4.80%, respectively) (Figure 4B,C).

### 2.2. In Silico Studies

#### 2.2.1. Molecular Docking

**Figure 5:**
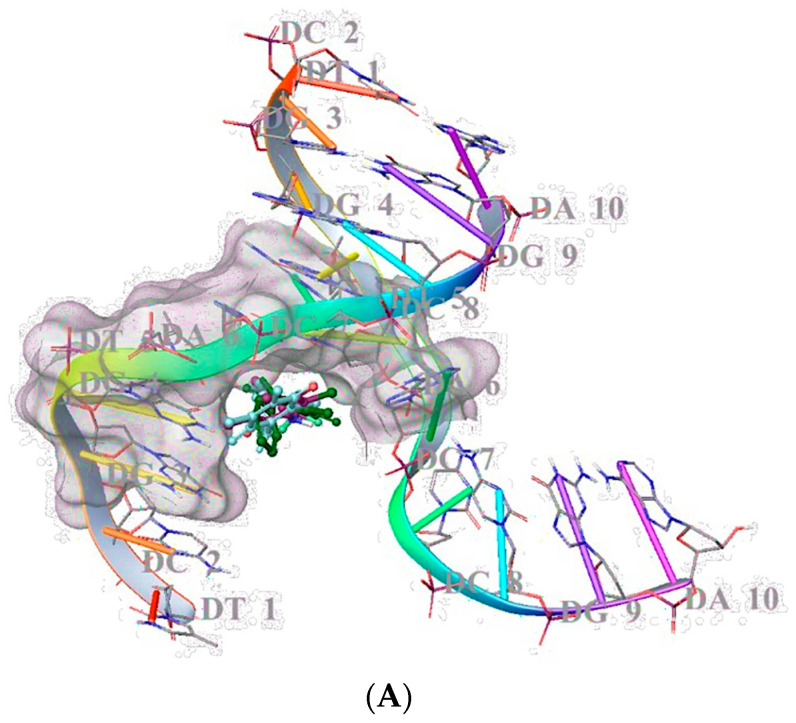

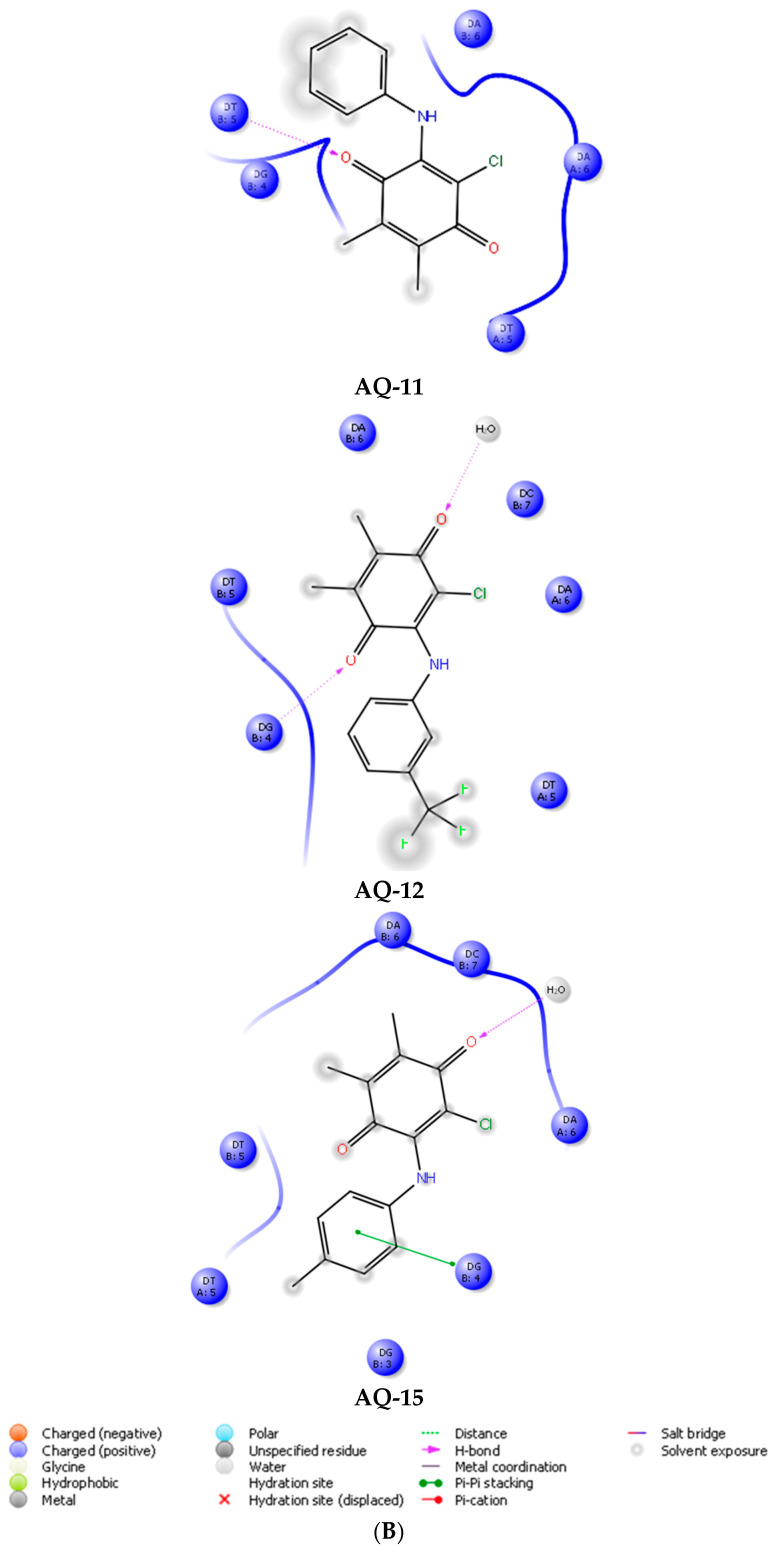
Docking poses (**A**) and docking interactions (**B**) of **AQ-11**, **AQ-12**, and **AQ-15** in the minor groove of the DNA double helix (**AQ-11**, **AQ-12**, and **AQ-15** are shown in dark green, maroon, and turquoise, respectively) (PDB ID: 2GWA).

In our previous studies, we manifested that PQ analogues were able to bind DNA significantly [26,27,42]. In this study, the DNA binding effects of **AQ-11**, **AQ-12**, and **AQ-15** were also searched with molecular docking studies in the minor groove of the double helix of DNA (PDB ID: 2GWA) [43] via Maestro software [44]. Results corresponded to previous DNA cleavage outcomes, implying that **AQ-15** showed the most promising DNA binding potential through a key π-π interaction between DG-4 with its 4-methyl substituent. However, **AQ-11** and **AQ-12** displayed less binding capacity compared to **AQ-15,** forming hydrogen bonding with DT-5 and DG-4, respectively (Figure 5A,B). The docking scores were determined as −4.641 kcal/mol, −5.087 kcal/mol, and −5.097 kcal/mol for **AQ-11**, **AQ-12**, and **AQ-15,** indicating higher binding capacity of **AQ-15** compared to **AQ-11** and **AQ-12**.

#### 2.2.2. Estimation of Pharmacokinetic Parameters

**AQ-12** was profiled *in silico* for various pharmacokinetic properties of interest such as octanol/water partition coefficient (QPlogPo/w), aqueous solubility (QPlogS), human serum albumin binding (QPlogKhsa), brain/blood partition coefficient (QPlogBB), and compliance to Lipinski’s rule of five and Jorgensen’s rule of three using the QikProp algorithm [45]. We also checked the *in silico* inhibitory potential of **AQ-12** on several cytochrome P450 (CYP) enzymes such as CYP1A2, CYP2C19, CYP2C9, CYP2D6, and CYP3A4, along with the evaluation of bioavailability and passive gastrointestinal absorption and brain penetration using the SwissADME web service [46,47].

**AQ-12** represented a remarkable pharmacokinetic profile in which all the descriptors were found in appropriate ranges: QPlogPo/w, QPlogS, QPlogKhsa, and QPlogBB were computed with the values of 3.513, −5.035, 0.197, and 0.140, respectively, within the limits (−2 to 6.5, −6.5 to 0.5, −1.5 to 1.5, and −3 to 1.2, respectively). Additionally, **AQ-12** revealed robust human oral absorption (100%) and was found to possess of all the conditions of drug-likeness characters without any violation of Lipinski’s rule of five and Jorgensen’s rule of three.

**Figure 6:**
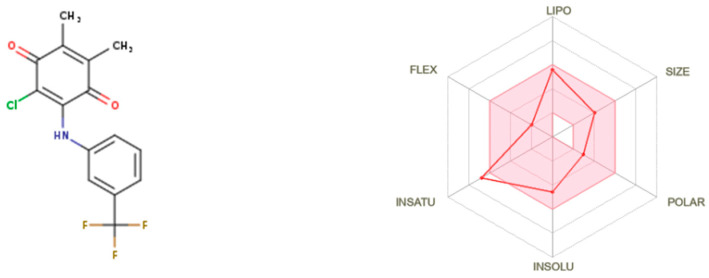
Bioavailability radar for **AQ-12** from the SwissADME web tool.

**Figure 7:**
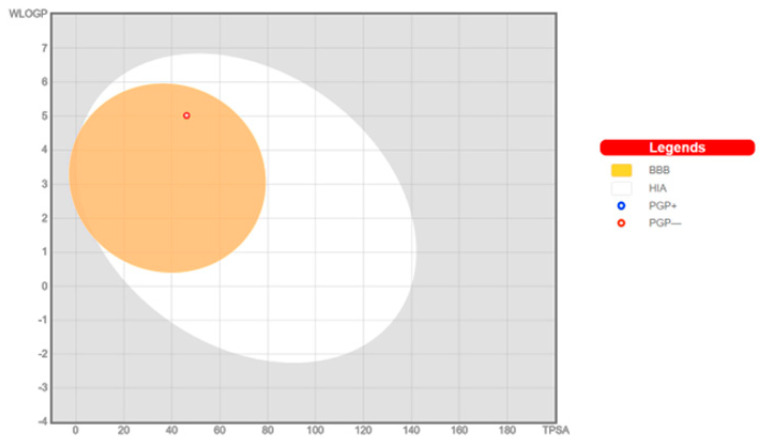
Boiled-egg graph of **AQ-12** from the SwissADME web tool.

The pink region of bioavailability radar (Figure 6) identifies the values of saturation (INSATU), size (SIZE), polarity (POLAR), solubility (INSOLU), lipophilicity (LIPO), and flexibility (FLEX) for oral bioavailability. **AQ-12** was found only beyond the saturation value for other values it was participating in, as shown in the pink area. **AQ-12** matched with CYP1A2, CYP2C19, CYP2C9, and CYP3A4 inhibition, apart from CYP2D6 inhibition, indicating that **AQ-12** could cause possible drug–drug or drug–food interactions. The boiled-egg model (Figure 7) explains whether a molecule has properties for the passive gastrointestinal absorption and blood–brain barrier (BBB) permeation. According to the results, **AQ-12** was predicted as brain-penetrant (in the yellow area) and not a substrate for P-glycoprotein (red dot), which decreased the possibility of its resistance by tumor cell lines through efflux [47,48,49,50].

## 3. Discussion

Worldwide, CRC and breast cancer are prevalent and deadly cancers. The complete cure for both cancers is still far from success, albeit to increase overall survival rate obtained with new therapeutic options. Therefore, the discovery of new and better therapeutics is still needed [51,52]. In spite of numerous efforts in the search for more effective anticancer agents, quinone moiety still remains one of the most versatile members against cancer cell lines in drug discovery [53,54].

**Figure 8:**
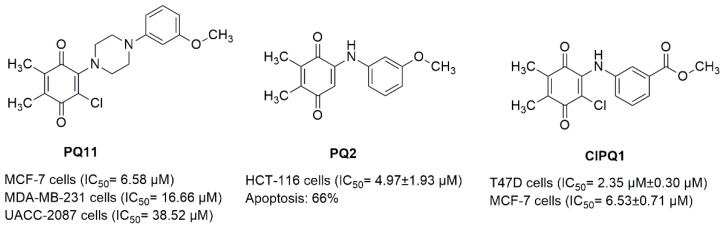
PQ analogues that were previously determined by our research group as potential anticancer agents against CRC and breast cancer.

In our previous studies, we also reported the significant outcomes of quinone derivatives against CRC or breast cancer cell lines. We showed that compound **PQ11**, PQ analogue with *N*-phenylpiperazine (Figure 8), exhibited the most potent anticancer activity against MCF-7, MDA-MB-231, and UACC-2087 cell lines, with the IC_50_ values of 6.58, 16.66, and 38.52 μM [28]. In our recently published studies, we also confirmed anti-CRC and anti-breast cancer effects of PQ analogues. In the first study [42], the most significant cytotoxic effects were observed with **PQ2**, amino-1,4-benzoquinone (Figure 8), against HCT-116 CRC cells with an IC_50_ value of 4.97 ± 1.93 μM. In the latter one [55], compound **ClPQ1**, quinone-benzocaine hybrid molecule, (Figure 8) was found as the most effective anti-breast cancer agent against T47D and MCF-7 breast cancer cells, with IC_50_ values of 2.35 ± 0.30 and 6.53 ± 0.71 μM, respectively. 

In the current work, PQ analogues (**AQ-11**, **AQ-12**, and **AQ-15**) were selected by the NCI *in vitro* disease-oriented antitumor screening to be evaluated for their anticancer effects. Testing of the PQ analogues against the NCI-60 cell line panel revealed valuable information on their inhibitory activity across a broad variety of human cancer cell lines. In particular, **AQ-12** displayed potential growth inhibitory activity against HCT-116 and MCF-7 cell lines at a single dose and a super-sensitivity profile with low micromolar GI_50_, TGI, and LC_50_ values against both cell lines at five doses. These findings indicated that meta trifluoromethyl substitution of **AQ-12** played an important role in its significant anti-CRC and anti-breast cancer activity when compared with the para methyl substitution of **AQ-15** and non-substitution of **AQ-11**. Moreover, **AQ-12** exerted similar cytotoxic effects against both cell lines in comparison with our aforementioned studies [28,42,55]. Current results once more confirmed that the presence of PQ moiety played an important role in anti-CRC and anti-breast cancer activity. 

Genetically encoded programmed cell death (apoptosis) leads to elimination of cancer cells, and DNA degradation is one of the crucial indicators of apoptosis. Aberrant apoptotic activity can increase not only the pathogenesis of CRC and breast cancer, but also their resistance to current therapy options [56,57,58,59]. Regarding the anticancer efficacy of **AQ-12** in CRC and breast cancer cells, it was ascertained that **AQ-12** led to apoptosis in both cells with similar apoptotic pattern with **PQ2** (Figure 8) [42].

Molecular docking studies were carried out for **AQ-12** in order to discover its binding efficacy in the minor groove of the double helix of DNA (PDB ID: 2GWA). We previously showed that PQ analogues occupied this region with key interactions [26,27,42]. The 3,5-dimethyl phenyl [26] and benzodioxole [27] moieties were determined to be crucial in binding with DNA, forming π-π stacking interactions with DA-17 and DG-16, and DA-5 and DG-4, respectively. In other our previous study [27], the methoxy substitution was also found to be important for high interaction between **PQ2** and DT-5 in the minor groove of DNA. In the current study, **AQ-12** was found less capable of binding DNA compared to **AQ-15**, albeit to hydrogen bonding with DG-4 through quinone moiety. The trifluoromethyl substitution of **AQ-12** played no significant role in binding with DNA. **AQ-15** bound to DG-4 through its *p*-methyl moiety, forming π-π stacking interactions. The docking score with the lowest energy (high negative scores) was found to pertain to **AQ-15,** followed by **AQ-12** and **AQ-11,** indicating their binding affinities. Compare to our previous studies, it can be concluded that CH_3_ substitution (−σ effect), OCH_3_ substitution (−σ effect), and (-CH_2_-O-CH_2_-) (−σ effect) [26,27,42] were found to increase the binding capacity of the tested compounds, whereas CF_3_ substitution (+σ effect) was not detected to contribute to binding capacity of **AQ-12**. The higher docking score and the binding capacity of *p*-methyl-substituted **AQ-15** also complied with the previous data. This finding also suggested that the high apoptotic effect of **AQ-12** might be independent from DNA cleavage-associated cell death.

Absorption, distribution, metabolism, and excretion (ADME) parameters of a drug molecule have an enormous impact for successful drug discovery. Some of these essential parameters were predicted *in silico* for **AQ-12**. Lipophilicity is crucial for absorption, which is the process of movement of a drug into the systemic circulation crossing the lipid bilayers of cell membranes. On the other hand, optimum water solubility is also necessary since the active ingredient must be dissolved in aqueous compartments to some extent before drug absorption. The human serum albumin binding is directly associated with the volume of distribution and half-life of drugs. The transition of drugs from blood into brain is particularly important for brain metastases of other cancer types. According to the results of the QikProp module, **AQ-12** was endowed with drug-like properties. The outcomes of SwissADME web server signified that **AQ-12** was predicted not orally bioavailable. This was due to the out-of-limits for saturation, as shown in the bioavailability chart, in which a molecule must be entirely included in the pink area. **AQ-12** exerted inhibition against all tested CYP enzymes, except for CYP2D6, which had a higher risk for drug–drug interactions [60,61,62,63].

## 4. Materials and Methods

### 4.1. Chemistry

The synthesis and spectral analysis of **AQ-11**, **AQ-12**, and **AQ-15** were performed previously [25].

### 4.2. Anticancer Activity Studies

#### 4.2.1. *In Vitro* Single-Dose Anticancer Screening by NCI

The PQ analogues were submitted to NCI, Bethesda, USA, and screened based on the procedures of NCI; all compounds were investigated for their cancer cell growth inhibitory activity at 10 µM concentration against a wide range of cancer cell lines stemming from leukemia, melanoma, CRC, non-small cell lung, CNS, ovarian, renal, prostate, and breast cancers. Tested compounds were added to the microtiter culture plates followed by incubation for 48 h at 37 °C. SRB was used for end point detection. The percent of growth of the treated cells was observed compared to the untreated control cells. Data from one-dose experiments corresponded to the percentage growth at 10 μM [29,30,31,34,64].

#### 4.2.2. *In Vitro* Five-Dose Anticancer Screening by NCI

Initial DMSO stock solution was carried out for serial 5 × 10-fold dilution before incubation at each individual concentration. **AQ-12** was selected for a higher testing level by DTP-NCI to identify GI_50_, TGI, and LC_50_ for each cell line after generating a dose response curve from 5 different concentrations (0.01, 0.1, 1, 10, and 100 µM). The definite protocol for the latter assay was explained in detail previously. The cells were assayed by using the SRB method. The optical densities were measured by a plate reader and a microcomputer processed the optical densities into the special concentration parameters, as defined above [29,34,64,65].

#### 4.2.3. Cell Culture, Drug Treatment, and MTT Assay

The HCT-116 cell line (provided by the RIKEN BRC through the National Bio-Resource Project of the MEXT/AMED, Japan (RCB2979)) and MCF-7 cell line (Precision Bioservices, Frederick, MD, USA) were incubated in Dulbecco’s modified Eagle’s medium (DMEM) (Wako Pure Chemical Industries, Osaka, Japan) and RPMI 1640 (Wako Pure Chemical Industries, Osaka, Japan), respectively. Ten percent fetal bovine serum (FBS) (Sigma Aldrich, St. Louis, MO, USA) and 89 μg/mL streptomycin (Meiji Seika Pharma, Tokyo, Japan) were added to total media (Wako Pure Chemical Industries) at 37 °C and 5% CO_2_ atmosphere. HCT-116 and MCF-7 cells were cultured for 48 h in a 24-well plate (Iwaki brand Asahi Glass Co., Chiba, Japan) at 4 × 10^4^ cells/mL concentration [42]. The stock solution of **AQ-12** and cisplatin in concentrations were prepared in DMSO (Wako Pure Chemical Industries, Osaka, Japan) (0.1 to 10 mM), and fresh culture medium was used for further dilution. The final DMSO concentration was set at 1% to prevent any effect of it on cell viability. MTT (Dojindo Molecular Technologies, Kumamoto, Japan) was used to examine the cytotoxic effects of **AQ-12** and cisplatin, as previously indicated [66]. HCT-116 and MCF-7 cells were treated with **AQ-12** and cisplatin at five dose concentrations (1, 3, 10, 30, and 100 μM) at 37 °C for 48 h, and then treated with MTT solution and incubated for 4 h. Eventually, 100 μL DMSO was added to each well following removal of supernatants. Infinite M1000 (Tecan, Mannedorf, Switzerland) was used for the analysis of the absorbance of the solution. All experiments were repeated three times, and IC_50_ values were calculated as the drug concentrations that diminished absorbance to 50% of control values.

#### 4.2.4. Cell Death Analysis

The HCT-116 and MCF-7 cell lines were incubated with **AQ-12** and cisplatin at IC_50_ concentration for 12 h before the apoptotic/necrotic/detection kit (PromoKine, Heidelberg, Germany) was applied, with some alterations to the manufacturer’s guidance [42]. HCT-116 and MCF-7 cells, treated with appropriate content including binding buffer and staining solution, were analyzed by an all-in-one fluorescence microscope, Biorevo Fluorescence BZ-9000 (Keyence, Osaka, Japan). Numbers of apoptotic, late apoptotic/necrotic, and necrotic cells were determined based on the staining with annexin V and ethidium homodimer III, as previously explained [66].

#### 4.2.5. Statistical Analyses

All results were reported as means ± SD. One-way analysis of variance was used for the analysis of data. Differences were defined as significant at * *p* < 0.05, ** *p* < 0.01, and *** *p* < 0.001. GraphPad Prism7 (GraphPad Software, San Diego, CA, USA) was used for the determination of the IC_50_ values.

### 4.3. In Silico Studies

#### 4.3.1. Molecular Docking

**AQ-11, AQ-12**, and **AQ-15** were prepared with energy minimization by applying the OPLS_2005 force field at physiological pH using the LigPrep module. The crystallographic structure of DNA was downloaded from the PDB server (PDB ID: 2GWA) [43,44] and prepared for the docking assessment by the PrepWizard module of Maestro. Then, the determined grid by Grid Generation was used for molecular docking with Glide/XP docking procedures [26,27,42].

#### 4.3.2. ADME Prediction

The pharmacokinetic determinants of **AQ-12** were estimated by QikProp [45] and SwissADME web tool [46,47].

## 5. Conclusions

Our previous encouraging anticancer activity results obtained from PQ analogues guided us to analyze **AQ-11**, **AQ-12**, and **AQ-15** for further effects toward a wide spectrum of cancer cells by NCI-60 *in vitro* screening. **AQ-12** presented a promising growth inhibition in HCT-116 and MCF-7 cells at a single dose and submicromolar level anticancer activity at five doses. It was established that these two cell lines were found susceptible for further anticancer activity studies. The MTT assay outcomes also corresponded to notable anti-CRC and anti-breast cancer activity of **AQ-12** at different concentrations compared to cisplatin. This compound also enhanced apoptosis in both cell lines. Taken together, **AQ-12** could serve as a valuable lead molecule for CRC and breast cancer treatment with orally bioavailable favorable drug-like features.

## Figures and Tables

**Table 1:** The cytotoxic effects of **AQ-11**, **AQ-12**, and **AQ-15** on K562 cells and PBMCs in comparison with imatinib [25].

Compound	Substitution Groups	Cell Type (IC_50_, μM)		
	R	K562 ^a^	PBMC ^a^	SI ^c^
**AQ-11**	H	0.75 ± 0.05	5.14 ± 1.76	6.85
**AQ-12**	*m*-CF_3_	0.88 ± 0.06	3.00 ± 1.22	3.41
**AQ-15**	*p*-CH_3_	0.76 ± 0.04	7.64 ± 1.58	10.05
Imatinib ^b^		5.58 ± 1.83	33.92 ± 4.19	6.08

^a^ Cell lines include K562 leukemia cells and peripheral blood mononuclear cells (PBMC). ^b^ Used as a reference. ^c^ selectivity index (SI) = IC_50_ for PBMCs/IC_50_ for K562 cells.

**Table 2:** Anticancer activity results as per single-dose assay at 10 µM concentration as percent cell growth of **AQ-11, AQ-12**, and **AQ-15** after 48 h, based on Sulforhodamine B (SRB) assay at NCI.

Panel/Cancer Cell Line	Compound		
	AQ-11	AQ-12	AQ-15
	Growth Percentage of Cell Lines in NCI 60		
**Leukemia**			
CCRF-CEM	5.28	5.90	47.98
HL-60(TB)	45.64	38.46	73.23
K-562	15.93	3.78	54.03
MOLT-4	12.72	23.53	66.29
RPMI-8226	53.63	−3.12	87.67
SR	62.86	24.97	79.88
**Non-Small Cell Lung Cancer**			
A549/ATCC	99.81	96.78	101.90
EKVX	55.24	49.44	93.61
HOP-62	92.60	90.98	89.82
HOP-92	46.02	48.86	95.70
NCI-H226	96.94	90.87	101.59
NCI-H23	ND	ND	ND
NCI-H322M	99.29	100.88	104.93
NCI-H460	95.74	94.85	100.91
NCI-H522	83.46	17.73	93.90
**CRC**			
COLO 205	107.07	101.80	111.35
HCC-2998	ND	ND	ND
HCT-116	79.11	33.86	95.79
HCT-15	94.19	65.71	101.63
HT29	99.13	103.04	104.47
KM12	78.34	67.20	100.25
SW-620	96.49	17.07	102.49
**CNS Cancer**			
SF-268	95.39	94.34	95.65
SF-295	102.14	96.20	107.03
SF-539	95.42	93.40	97.15
SNB-19	88.79	92.41	96.00
SNB-75	65.44	66.47	65.44
U251	84.30	78.99	99.94
**Melanoma**			
LOX IMVI	ND	ND	ND
MALME-3M	107.98	94.35	102.03
M14	95.87	85.23	102.50
MDA-MB-435	102.30	98.46	107.59
SK-MEL-2	85.48	85.77	95.31
SK-MEL-28	106.11	98.26	104.07
SK-MEL-5	ND	ND	ND
UACC-257	90.27	78.53	106.17
UACC-62	90.28	80.22	96.80
**Ovarian Cancer**			
IGROV1	9.69	−7.20	80.83
OVCAR-3	101.79	70.40	105.64
OVCAR-4	−97.92	−80.41	99.38
OVCAR-5	103.60	100.01	100.88
OVCAR-8	94.89	87.14	104.55
NCI/ADR-RES	ND	ND	ND
SK-OV-3	ND	ND	ND
**Renal Cancer**			
786-0	99.18	101.74	104.29
A498	91.24	55.17	72.19
ACHN	100.46	−37.69	96.49
CAKI-1	96.08	92.74	91.53
RXF 393	96.80	97.82	108.41
SN12C	91.13	87.62	96.37
TK-10	128.28	183.73	142.33
UO-31	98.81	77.33	92.50
**Prostate Cancer**			
PC-3	73.87	64.35	86.95
DU-145	101.26	99.72	107.98
**Breast Cancer**			
MCF7	90.72	35.36	96.29
MDA-MB-231/ATCC	15.35	18.96	94.55
HS 578T	100.91	87.57	90.46
BT-549	113.97	120.60	119.94
T-47D	−38.88	−38.96	84.68
MDA-MB-468	−76.01	−65.55	52.82

**Table 3:** GI_50_, TGI, and LC_50_ values (in µM) of anticancer activity data, as per five doses (0.01, 0.1, 1, 10, and 100 µM) of **AQ-12** after 48 h based on SRB assay at NCI.

Panel/Cell Line	GI_50_	TGI	LC_50_
**Leukemia**			
CCRF-CEM	1.93	>100	>100
HL-60(TB)	2.34	6.54	>100
K-562	2.40	>100	>100
MOLT-4	2.22	>100	>100
RPMI-8226	1.32	7.32	>100
SR	2.59		>100
**Non-Small Cell Lung Cancer**			
A549/ATCC	13.30	>100	>100
EKVX	1.49	3.73	9.31
HOP-62	11.50	27.60	66.50
HOP-92	1.51	3.04	6.14
NCI-H226	10.80	34.90	>100
NCI-H23	6.87	24.00	65.40
NCI-H322M	22.70	>100	>100
NCI-H460	11.60	32.20	89.30
NCI-H522	2.24	5.66	>100
**CRC**			
COLO 205	13.00	29.30	66.00
HCC-2998	5.88	18.40	46.00
HCT-116	1.93	3.99	8.22
HCT-15	2.20	5.05	16.20
HT29	7.29	51.80	>100
KM12	3.58	25.70	>100
SW-620	2.09	4.73	>100
**CNS Cancer**			
SF-268	4.99	28.60	>100
SF-295	6.55	24.00	74.10
SF-539	2.40	6.61	24.80
SNB-19	5.83	23.40	77.40
SNB-75	1.16	13.60	91.00
U251	4.45	17.90	58.20
**Melanoma**			
LOX IMVI	1.69	3.32	6.51
MALME-3M	1.98	6.04	30.90
M14	9.96	32.70	>100
MDA-MB-435	5.98	19.50	49.20
SK-MEL-2	8.26	21.70	50.90
SK-MEL-28	3.36	10.80	36.00
SK-MEL-5	4.14	15.70	43.80
UACC-257	2.61	6.72	26.90
UACC-62	3.46	16.10	56.00
**Ovarian Cancer**			
IGROV1	1.42	3.19	7.15
OVCAR-3	2.31	5.58	>100
OVCAR-4	1.58	2.97	5.58
OVCAR-5	1.98	4.13	8.61
OVCAR-8	2.68	6.89	>100
NCI/ADR-RES	3.16	42.30	>100
SK-OV-3	1.30	50.70	>100
**Renal Cancer**			
786-0	10.60	24.30	55.70
A498	13.70	45.20	>100
ACHN	1.73	3.19	5.87
CAKI-1	1.94	9.11	32.40
RXF 393	1.49	2.85	5.44
SN12C	2.25	23.40	>100
TK-10	19.00	37.00	71.90
UO-31	1.46	4.18	14.20
**Prostate Cancer**			
PC-3	2.67	12.80	>100
DU-145	15.60	34.80	77.70
**Breast Cancer**			
MCF7	1.71	3.79	8.39
MDA-MB-231/ATCC	1.59	3.28	6.77
HS 578T	6.00	64.40	>100
BT-549	8.56	22.20	51.70
T-47D	1.17	3.60	>100
MDA-MB-468	1.24	2.94	7.00

**Table 4:** The cytotoxic effects of **AQ-12** on MCF-7 and HCT-116 cells based on MTT assay at five dose concentrations (1, 3, 10, 30, and 100 μM).

Compound	IC_50_ Value (µM)	
	MCF-7 Cells	HCT-116 Cells
**AQ-12**	6.06 ± 3.09	5.11 ± 2.14
**Cisplatin**	19.67 ± 5.94	23.68 ± 6.81

## Data Availability

Not applicable.

## Supplementary Materials

The following supporting information can be downloaded at: https://www.mdpi.com/article/10.3390/ph15101266/s1, Figure S1: One-dose mean graph of **AQ-11** against different cancer cell lines based on Sulforhodamine B (SRB) assay at NCI at 10 µM concentration after 48 h; Figure S2: One-dose mean graph of **AQ-12** against different cancer cell lines based on SRB assay at NCI at 10 µM concentration after 48 h; Figure S3: One-dose mean graph of **AQ-15** against different cancer cell lines based on SRB assay at NCI at 10 µM concentration after 48 h.
