# Supplementary material for: In Vitro Cytotoxicity Evaluation of Plastoquinone Analogues against Colorectal and Breast Cancers along with In Silico Insights
Source: Pharmaceuticals (Basel). 2022 Oct 14;15(10):1266. doi: 10.3390/ph15101266 (PMC9609592; doi:10.3390/ph15101266)
Supplement: Supplementary file 1 [file pharmaceuticals-15-01266-s001.zip › pharmaceuticals-1935459-supplementary.pdf]

Supplementary Material (SM)

In Vitro Cytotoxicity Evaluation of Plastoquinone Analogues against Colorectal and Breast Cancers along with *In Silico* Insights

Halilibrahim Ciftci [1,2,3], Belgin Sever [2,4], Nilüfer Bayrak [5], Mahmut Yıldız [6], Hatice Yıldırım [7], Hiroshi Tateishi [2], Masami Otsuka [1,2], Mikako Fujita [2,*] and Amaç Fatih TuYuN [5,*]

[1] Department of Drug Discovery, Science Farm Ltd., Kumamoto 862-0976, Japan
[2] Medicinal and Biological Chemistry Science Farm Joint Research Laboratory, Faculty of Life Sciences, Kumamoto University, Kumamoto 862-0973, Japan
[3] Department of Molecular Biology and Genetics, Koc University, Istanbul 34450, Turkey
[4] Department of Pharmaceutical Chemistry, Faculty of Pharmacy, Anadolu University, Eskisehir 26470, Turkey
[5] Department of Chemistry, Faculty of Science, Istanbul University, Fatih, Istanbul 34126, Turkey
[6] Chemistry Department, Gebze Technical University, Gebze, Kocaeli 41400, Turkey
[7] Department of Chemistry, Faculty of Engineering, Istanbul University-Cerrahpasa, Avcılar, Istanbul 34320, Turkey
* Correspondence: mfujita@kumamoto-u.ac.jp (M.F.); aftuyun@istanbul.edu.tr (A.F.T.); Tel.: +81-96-371-4622 (M.F.); +90-212-4400000 (A.F.T.)

One-dose mean graphs of **AQ-11**, **AQ-12**, and **AQ-15** against different cancer cell lines.

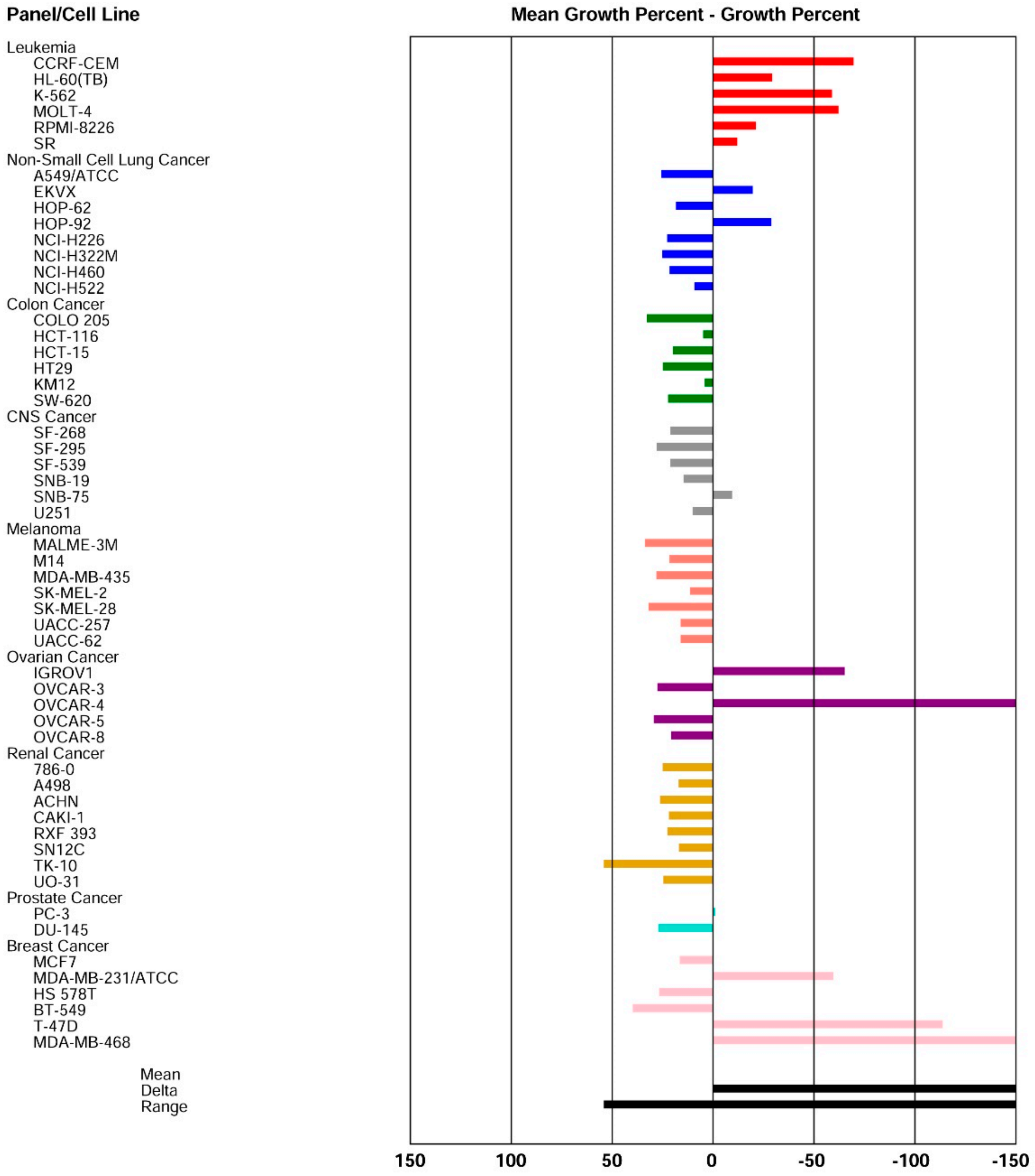

Figure S1. One-dose mean graph of **AQ-11** against different cancer cell lines based on Sulforhodamine B (SRB) assay at NCI at 10 μM concentration after 48 h.

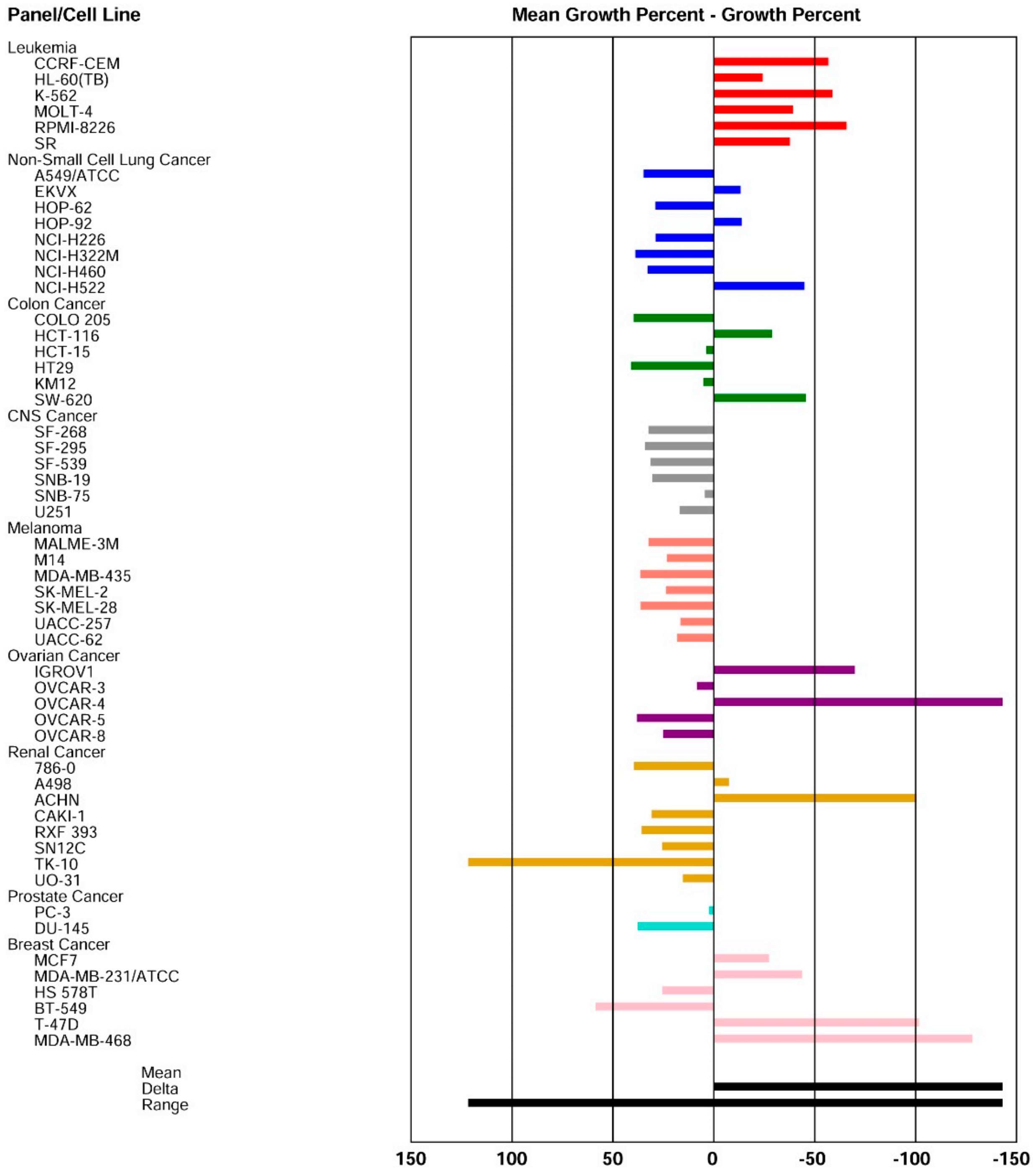

Figure S2. One-dose mean graph of **AQ-12** against different cancer cell lines based on SRB assay at NCI at 10 μM concentration after 48 h.

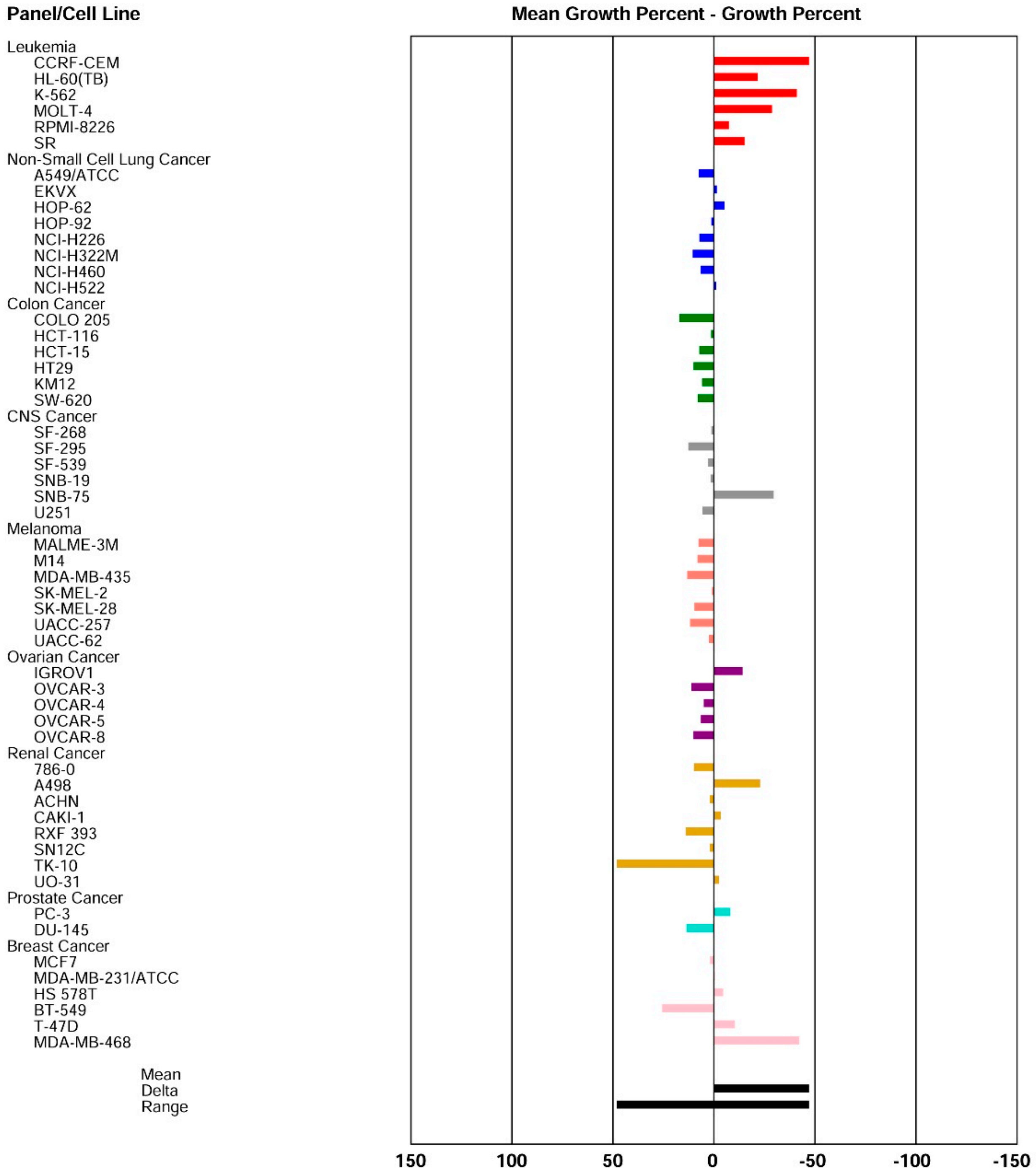

Figure S3. One-dose mean graph of **AQ-15** against different cancer cell lines based on SRB assay at NCI at 10 μM concentration after 48 h.
